# Contractile effects of albiglutide in the human and mouse atrium

**DOI:** 10.3389/fendo.2026.1831742

**Published:** 2026-07-03

**Authors:** Joachim Neumann, Milena Jarikova, Uwe Kirchhefer, Britt Hofmann, Ulrich Gergs

**Affiliations:** 1 Institute for Pharmacology and Toxicology, Medical Faculty, Martin-Luther-University Halle-Wittenberg, Halle (Saale), Germany; 2 Institute for Pharmacology and Toxicology, Medical Faculty, University Münster, Münster, Germany; 3 Department of Cardiac Surgery, Mid-German Heart Centre, University Hospital Halle, Halle (Saale), Germany

**Keywords:** albiglutide, GLP-1 receptor (GLP-1R), human atrium, INOTROPY, phospholamban (PLB)

## Abstract

Albiglutide was developed as a glucagon-like-peptide 1 receptor (GLP-1R) agonist for the treatment of type 2 diabetes. We tested the hypothesis that albiglutide exerts a positive inotropic effect in the human heart via GLP-1R by measuring contractility in paced (1 Hz) human atrial preparations (HAP) from adult patients who underwent surgery for severe coronary heart disease. We observed a time- and concentration-dependent positive inotropic effect of albiglutide in HAP; this effect began at 10 nM albiglutide and increased to 100 nM albiglutide, the highest concentration studied. The positive inotropic effect of albiglutide was accentuated by the phosphodiesterase III inhibitor cilostamide (100 nM) and reduced by 10 µM N-[2-[[(E)-3-(4-bromophenyl)prop-2-enyl]amino]ethyl]isoquinoline-5-sulfonamide, an inhibitor of the 3',5' cyclic adenosine monophosphate-dependent protein kinase. Conversely, the effect was not reversed by 10 µM propranolol, a β-adrenoceptor antagonist, and was less effective than the effect of 1 µM isoprenaline. In the presence of cilostamide, the endogenous agonist GLP-1(7-36)amide at 100 nM augmented the force of contraction in HAP, whereas its precursor, GLP-1(1-36)amide, did not. Contractile force in abligutide-treated (100 nM) HAP in the presence of cilostamide was further enhanced by 100 nM GLP-1(7-36)amide or 10 nM exenatide but was not affected by 100 nM GLP-1(1-36)amide. After the addition of cilostamide, 100 nM albiglutide augmented the rate of tension relaxation and accelerated the time to relaxation in HAP. In contracting HAP, 100 nM albiglutide in the presence of 100 nM cilostamide increased the phosphorylation state of phospholamban and the inhibitory subunit of troponin. In HAP, the positive inotropic effects of albiglutide—both alone and in the presence of 100 nM cilostamide—were attenuated by treatment with 100 nM exendin(9-39), a GLP-1R antagonist. In contrast, 100 nM albiglutide did not increase the force of contraction or the beating rate in isolated left atrial or right atrial preparations from adult mice in the presence and absence of the phosphodiesterase 4 inhibitor rolipram (100 nM). Our data suggest that albiglutide increases the force of contraction via stimulation of GLP-1R and cAMP-dependent phosphorylation in HAP. Albiglutide acts as a partial GLP-1R agonist in HAP. This indicates that any clinical side effects of albiglutide may be less pronounced than those of full GLP-1R agonists.

## Introduction

**Figure 1:**
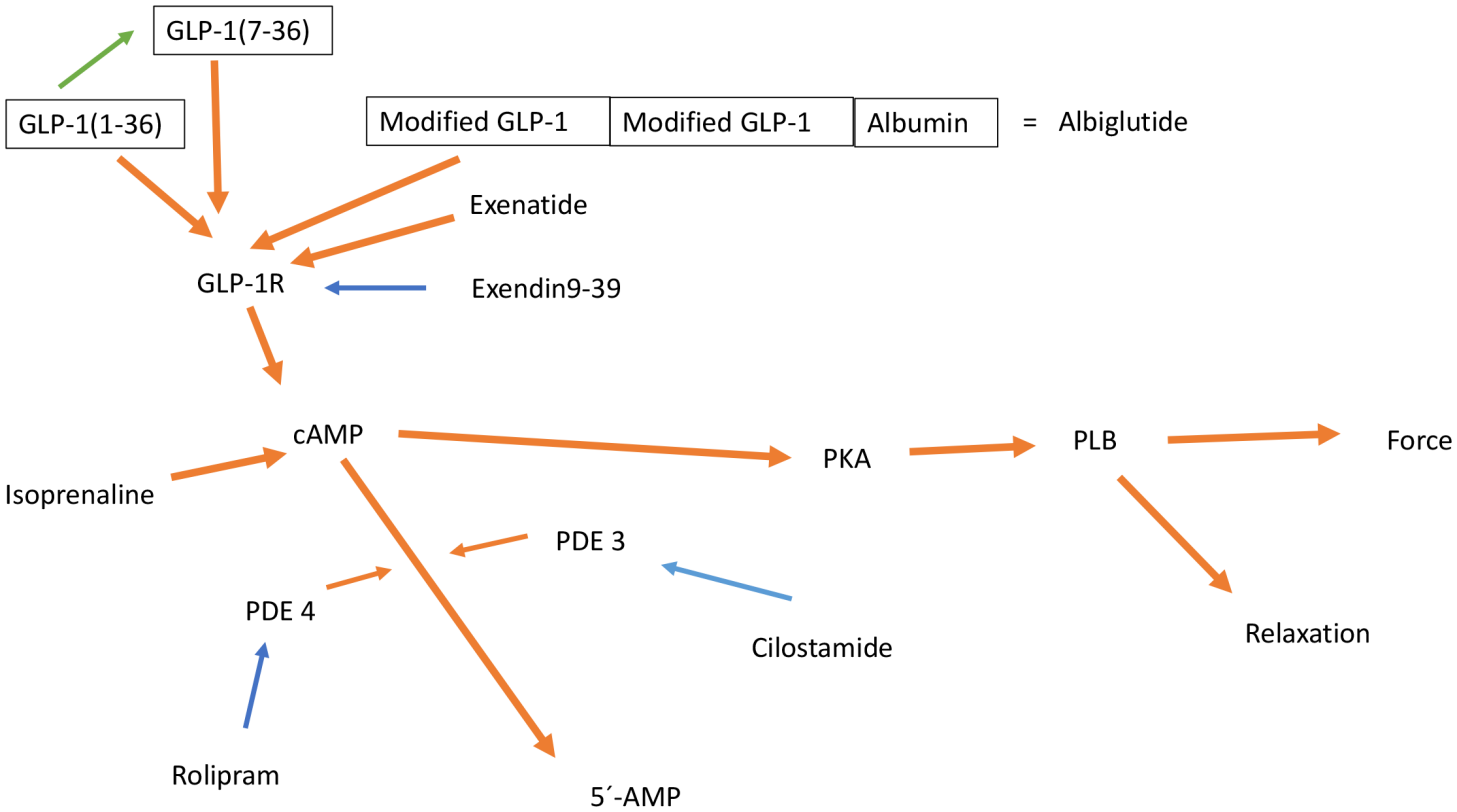
Mechanism(s) of action of albiglutide in cardiomyocytes. Endogenous glucagon-like-peptide 1 (GLP-1) stimulates (red arrow) the GLP-1 receptor (GLP-1R) on the sarcolemma, resulting in cAMP generation. GLP-1 is initially formed as GLP-1(1-36)amide (GLP-1(1-36)). Proteases (green arrow) generate GLP-1(7-36) amide (GLP-1(1-37)), which is a more potent agonist of GLP-1R. The GLP-1R is antagonised (blue arrow) by exendin(9-39). Isoprenaline also elevates cAMP levels. Subsequently, cAMP activates cAMP-dependent protein kinase (PKA). PKA phosphorylates and activates phospholamban (PLB). PLB enhances cardiac contractile force and promotes muscle relaxation. cAMP is degraded by phosphodiesterases (PDE) to inactive 5′-AMP. PDE3 and PDE4 are inhibited (blue arrows) by cilostamide and rolipram, respectively. The N-terminus of albiglutide contains a modified GLP-1 peptide linked to human albumin via a second modified GLP-1 peptide.

Glucagon-like-peptide 1 (GLP-1) activates the GLP-1 receptor (GLP-1R, **Figure 1**) in the pancreas. This activation results in the release of insulin and inhibits the release of glucagon. As a result, blood glucose levels decline. This forms the basis for the use of GLP-1R agonists for the treatment of type 2 diabetes (1, 2). Initially, the precursor GLP-1(1-36)amide is formed in the gut (3, 4). A protease cleaves the first six amino acids to form the active metabolite GLP-1(7-36)amide (**Figure 1**). The first clinically approved GLP-1R agonist was exenatide. Exendin-4 (=exenatide), a peptide from the saliva of lizards, has the advantage of a much longer half-life than GLP-1; however, it still requires administration by injection once or twice daily in patients (5). Several drugs based on the protein sequence of GLP-1 but with longer half-lives than exenatide were developed to activate GLP-1R in the pancreas (6). This led to the development of albiglutide (7), which was approved for the treatment of type 2 diabetes. Albiglutide has beneficial effects on cardiovascular morbidities in patients (8–12). Albiglutide features two mutated copies of GLP-1 at its amino-terminal end. These mutations are single amino acid exchanges: at position eight, alanine is replaced by glycine (**Figure 1**). This modification was introduced to reduce degradation of GLP-1 by dipeptidyl peptidase-4. The carboxyterminal end of albiglutide contains albumin, which is intended to confer additional stability against proteases. Albiglutide exhibits a half-life of 6 days (13). GLP-1R belongs to the family of heptahelical receptors (**Figure 1**), which also includes β-adrenoceptors. Similar to β-adrenoceptors, GLP-1R signals via stimulatory guanosine triphosphate-binding proteins that stimulate adenylyl cyclase, thereby increasing intracellular cAMP levels in GLP-1R-transfected cells (**Figure 1**) (1). Exenatide was found to be more potent and effective than albiglutide in increasing cAMP levels in baby hamster cells transfected with rat GLP-1R (14). Therefore, in this study, we compared albiglutide with other GLP-1R agonists, including exenatide and the endogenous GLP-1 peptide. Exenatide has also been shown to increase the force of contraction in isolated human atrial preparations (HAP; 15). Similarly, semaglutide—another GLP-1R agonist—exerted a positive inotropic effect in HAP or in human isolated left ventricular muscle strips (16–18). Liraglutide, also a GLP-1 agonist, increased contractility in isolated HAP (16). The effects of exenatide, liraglutide, and semaglutide on the force of contraction were attenuated by the GLP-1R antagonist exendin(9-39) (15–18). Moreover, semaglutide (300 nM) elevated cAMP levels in isolated HAP and Ca^2+^ transient amplitudes in human ventricular preparations isolated from patients with heart failure (17, 18). There is evidence that semaglutide reduces isoprenaline-induced arrhythmias in HAP (18). Moreover, exenatide increased the phosphorylation state of phospholamban at serine 16—the phosphorylation site for the cAMP-dependent protein kinase—in HAP (15). Semaglutide increased the Ca^2+^ load in the sarcoplasmic reticulum of HAP (18), which can be explained by increased activity of sarcoplasmic/endoplasmic reticulum calcium ATPase (SERCA), caused by the phosphorylation of phospholamban (19). However, to the best of our knowledge, there is no previous research on whether albiglutide could augment the force of contraction in HAP and, if so, through which mechanism this occurs. Regarding semaglutide, there are clinical data indicating that it can have beneficial effects in patients with heart failure with preserved ejection fraction (20). This benefit is also observed in patients without diabetes (21). Thus, it has been speculated that semaglutide might act through mechanisms other than reducing blood glucose levels (21). In addition, semaglutide may have direct beneficial effects on the human heart, either by reducing atrial and ventricular arrhythmias and/or by providing inotropic support in heart failure (17). Such effects may be agonist-dependent rather than class effects; for example, exenatide was associated with increased arrhythmias in study patients (22). Therefore, it may be helpful to study the inotropic effects of albiglutide in HAP and investigate agonist-dependent differences. Albiglutide rapidly crosses the blood–brain barrier (23). Looking ahead, this raises the possibility that albiglutide could be used in the treatment of Alzheimer’s disease and Parkinson’s disease, where GLP-1R agonists have shown promise (24).

Albiglutide has been tested in type 2 diabetes mouse models and in mice with obesity (e.g., 14, 23, 25, 26). Hence, it is of interest to determine whether albiglutide acts acutely on the force of contraction or beating rate in mouse hearts.

Thus, in this study, we tested the hypotheses that albiglutide increases contractile parameters in HAP or mouse left atrial preparations and increases the beating rate in mouse right atrial preparations.

## Materials and methods

### Contractile studies in mice

The mice were treated in compliance with local animal protection requirements (Permit Number I9M8, Veterinäramt der Stadt Halle, Halle [Saale], Germany). No animal experiments were performed, and only heart removal was carried out. Published procedures were used for these mice (16). In brief, the mice were euthanised via rapid cervical dislocation without anaesthesia, as required by the local ethical committee for animal research. The sternum was cut, and the heart was removed. Right and left atrial preparations from the hearts of wild-type mice (CD-1, both sexes, approximately 180 days old, bred in our institution) were isolated and mounted in organ baths containing modified Tyrode’s solution as the bathing solution: 119.8 mM NaCI, 5.4 mM KCI, 1.8 mM CaCl_2_, 1.05 mM MgCl_2_, 0.42 mM NaH_2_PO_4_, 22.6 mM NaHCO_3_, 0.05 mM Na_2_EDTA, 0.28 mM ascorbic acid, and 5.05 mM glucose. The solution was continuously gassed with 95% O_2_ and 5% CO_2_ (carbogen) and maintained at 37 °C and pH 7.4. In spontaneously beating right atrial preparations, chronotropic and inotropic effects were measured. Electrically stimulated (1 Hz) left atrial preparations from mice were used to measure inotropic effects under isometric conditions. The duration of electrical stimulation with a rectangular direct-current impulse was 5 ms. The voltage was set 10% higher than the threshold to initiate contraction. Drugs were pipetted directly into the organ baths (10 mL volume, heated to 37 °C). The custom-made organ bath contained a glass frit at the bottom, which allowed small bubbles of carbogen to enter the buffer and rise from the bottom to the surface of the organ bath. This facilitated the rapid mixing of any drug added to the organ bath. Details of drug additions are provided in the appropriate figure legends.

### Contractile studies on human preparations

Contractile studies on HAP were performed using the same setup and buffer described in the previous section (e.g., 16). In brief, the force of contraction was quantified in electrically paced (1 Hz) isolated right atrial preparations. Samples were obtained from 14 patients (four female patients). The patients suffered from coronary diseases (two- and three-vessel disease), hypertension, and atrial fibrillation as the main cardiac morbidities. **Table 1** presents the characteristics of the patients. Informed written consent was obtained from all patients included in the study. None of the study patients received a GLP-1R agonist (**Table 1**). The study was approved by the local ethical commission (internal code: hm-bü). In some experiments, 10 µM propranolol was first added to block β-adrenoceptors for 10 min, followed by the addition of 100 nM cilostamide for 10 min. Thereafter, albiglutide was added to the organ bath. In separate experiments, 10 µM H89, an inhibitor of cAMP-dependent protein kinase, was first added for 10 min, followed by the addition of 100 nM cilostamide for 10 min. Thereafter, albiglutide was added to the organ bath. In some experiments, phosphodiesterase inhibitors were used to unveil or at least accentuate the contractile effects of albiglutide. Phosphodiesterase 4 is the main phosphodiesterase in the mouse heart, whereas phosphodiesterase 3 is the main phosphodiesterase in the human heart. Phosphodiesterase 4 is inhibited by rolipram and not cilostamide (27, 28). In contrast, phosphodiesterase 3 is not inhibited by rolipram but is inhibited by cilostamide (28). For these reasons, we used different phosphodiesterase inhibitors in mice and humans: rolipram was used in the relevant contraction studies in mice, whereas cilostamide was used in HAP.

**Table 1:** Clinical data for the patients studied.

Variables	*N* = 14 patients
Age (mean ± SEM)	66 ± 3.0
Male sex	10
Left ventricular ejection fraction (mean ± SEM)	53% ± 6%
Hypertension	7
Atrial fibrillation	2
Diabetes	3
Coronary artery bypass surgery	12
Valve operation	2
β-Adrenoceptor antagonists	8
Angiotensin II receptor antagonists	4
Angiotensin-converting enzyme inhibitor	8
Angiotensin receptor–neprilysin inhibitors	1
Sodium/glucose cotransporter 2 inhibitors	2
Mineralocorticosteroid receptor antagonists	1
Diuretics	7
GLP-1R agonists	0

The cardiac relevant comorbidities and tsshe cardiac related medications are indicated.

Our right mouse atrial preparations were essentially complete auricles of the mouse heart. These auricles contained the sinus nodes, which explains why the right atria beat spontaneously and do not require any electrical stimulation. The left atrial preparations of mouse hearts did not contain the sinus node; thus, murine left atrial preparations had to be electrically stimulated. We did not study the complete right auricle of the human subjects. This is only possible during cardiac transplantation, wherein an isolated heart or isolated sinus node can beat spontaneously. We only used a small strip from the centre of each right auricle because a hole was cut for the insertion of a needle for extracorporeal circulation. These strips were electrically stimulated in this study. Therefore, we could not study any effects of drugs on the beating rate in HAP.

### Western blotting

When the contraction studies in human atrial preparations (see above) produced the intended inotropic effects, the samples were rapidly removed from the organ bath and transferred within less than 10 s into precooled 1.5 mL plastic test tubes. The tubes were subsequently immersed in a beaker filled with liquid nitrogen. In this way, all enzymatic processes were terminated. The homogenisation of the frozen samples, protein measurements, electrophoresis, primary and secondary antibody incubation, and quantification were performed according to previously published protocols (29). In brief, the frozen human atrial preparations were mechanically homogenised in a frozen SDS/bicarbonate solution to inhibit enzymatic processes. The samples were then allowed to thaw, and the protein content of each sample was determined. In each lane of a 20-lane gel, 20 μg of protein from these homogenates were loaded. Electrophoresis was performed using precast gels (Novex™ 4%–20% “Tris-Glycine Plus Midi Protein Gels”, Invitrogen, Thermo Fisher Scientific, Waltham, MA, USA). Thereafter, the proteins were electrically transferred into nitrocellulose membranes using a phosphate buffer. The nitrocellulose membranes were incubated with an anti-calsequestrin antibody (No. ab3516, Abcam, Cambridge, England), an antibody against phospholamban phosphorylated at serine 16 (No. A010–12 AP, Badrilla, Leeds, England), or an antibody against phosphorylated troponin inhibitor, TnI. Signals were detected using a commercial imager (Amersham ImageQuant 800, Cytiva, Freiburg im Breisgau, Germany).

### Data analysis

The data are presented as mean ± standard error. The Shapiro–Wilk test was used to confirm the normality of the data. Statistical significance was estimated using analysis of variance (ANOVA), followed by Bonferroni’s *t*-test or a paired *t*-test, as appropriate. A *p*-value ≤ 0.05 was defined as statistically significant. PrismGraphpad 9 was used for statistical analysis and data presentation.

### Drugs and materials

Albiglutide was obtained from MedChemExpress (local distributor: Hycultec, Beutelsbach, Germany). Exendin(9-39) was obtained from Thermo Scientific (local distributor: Th. Geyer, Renningen, Germany). GLP-1 derivatives were obtained from Bachem, Bubendorf, Switzerland. All other chemicals were of the highest commercially available purity grade. Deionised water was used throughout the experiments to prepare the organ bath buffer. Stock solutions of drugs were prepared fresh daily.

## Results

**Figure 2:**
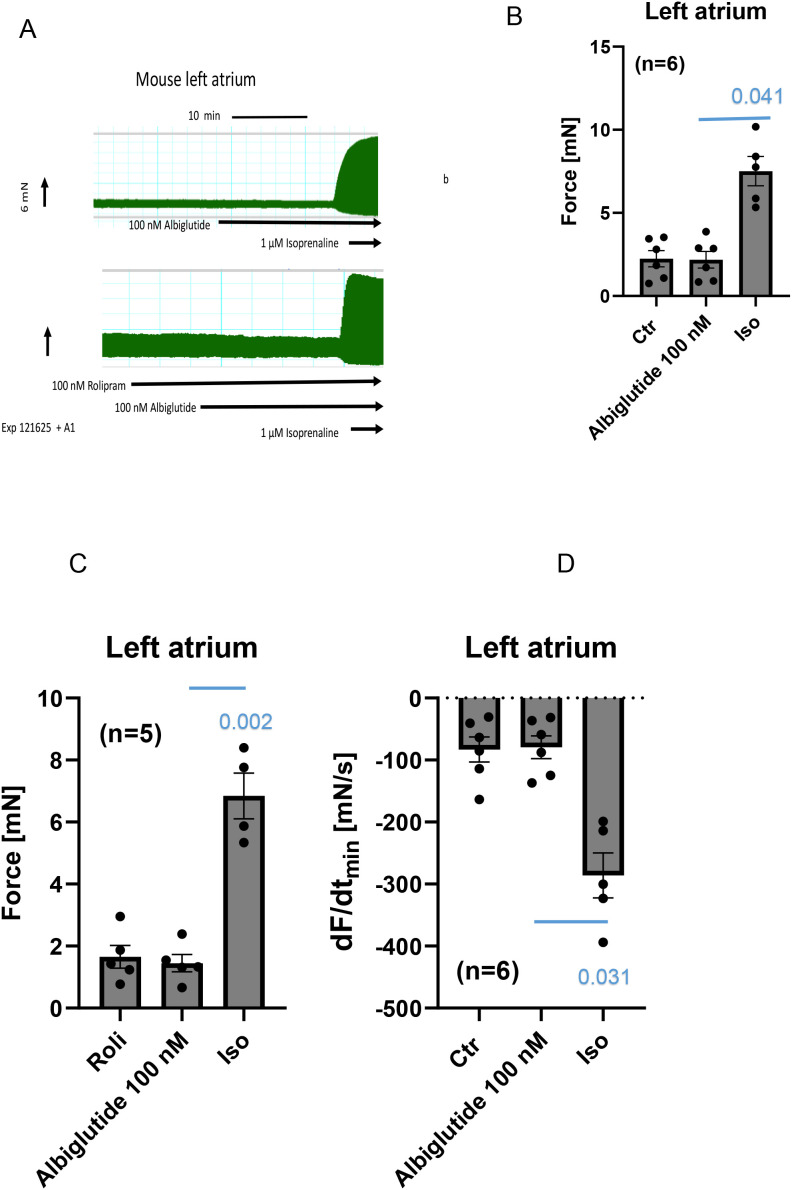
**(A)** Original recording of the effect of 100 nM albiglutide alone (top) or in the presence of rolipram (bottom) on force of contraction in mouse left atrial preparations. Subsequently, 1 µM isoprenaline (Iso) was added (bottom). Ordinates indicate force in milliNewtons (mN). Effects of albiglutide alone on the force of contraction and, for comparison, effects of 1 µM isoprenaline. Ordinates in **(A–C)** give the force of contraction in mN. Ordinate in **(D)** indicates the rate of muscle relaxation in mN per second (mN/s). Control (Ctr) indicates values before the addition of albiglutide. Rolipram (Roli) means after 100 nM rolipram and before albiglutide. Numbers in brackets indicate the number of experiments. Numbers (in blue) above or below the bars for Iso indicate *p*-values vs. albiglutide values.

We first present the results of the mouse atria experiments, followed by those from the human atria. We first examined whether albiglutide could increase the force of contraction in mouse left atrial preparations. However, albiglutide at concentrations up to 100 nM failed to increase the force of contraction in mouse left atrial preparations. This is shown in the original tracing depicted in **Figure 2A** (top). Moreover, in the presence of the phosphodiesterase 4 inhibitor rolipram (100 nM), albiglutide (100 nM) failed to increase the force of contraction (**Figures 2A**, bottom). We also added 1 µM isoprenaline as a positive control, as illustrated in **Figure 2** (bottom). This addition increased the force of contraction, demonstrating that the effects of cAMP-increasing agents could be measured under our experimental conditions. Several such experiments with albiglutide in the absence of rolipram are summarised in **Figure 2B**. Albiglutide did not increase the force of contraction, whereas isoprenaline augmented it (**Figure 2B**). Experiments with albiglutide in the presence of rolipram are shown in **Figure 2C** and demonstrate that, in the continued presence of rolipram, albiglutide failed to increase contractility, whereas, in the same left atria, isoprenaline increased contractility. Experiments on the effect of albiglutide alone on the rate of relaxation are summarised in **Figure 2D**. Albiglutide failed to increase the rate of relaxation. As expected, isoprenaline, used as a positive control, led to faster muscle relaxation (**Figure 2D**) (cf. 16, 30).

**Figure 3:**
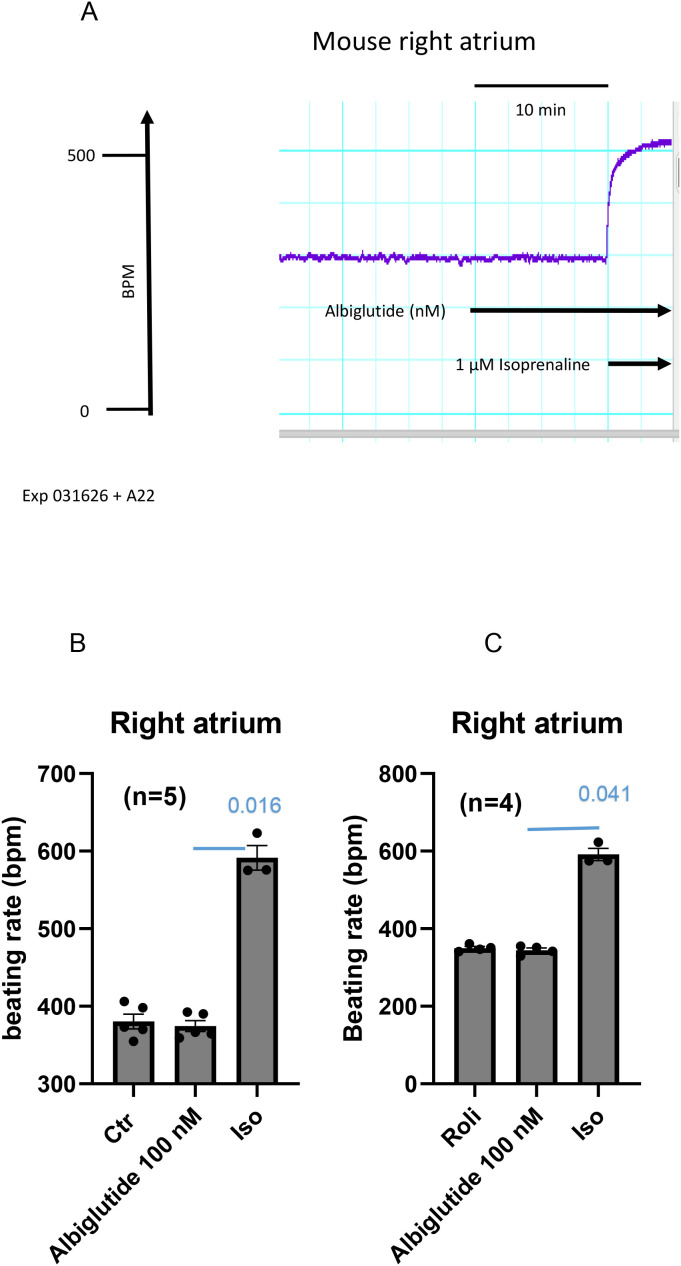
Original recording of the beating rate with 100 nM albiglutide in the mouse right atrial preparation **(A)**. Summarised effects of albiglutide alone **(B)** or in the presence of 100 nM rolipram **(C)** on beating rate, compared with 1 µM isoprenaline (Iso). Ordinates indicate beats per minute (bpm). Numbers in brackets indicate the number of experiments. Numbers (in blue) above or below the bars for Iso indicate *p*-values vs. albiglutide values.

Moreover, 100 nM albiglutide alone failed to elevate the beating rate in the right atria of mice, as shown in the original tracing, whereas subsequently applied 1 µM isoprenaline exerted a positive chronotropic effect (**Figure 3A**). Several such experiments are summarised in **Figure 3B**. Whereas albiglutide failed to increase the beating rate, the positive control, 1 µM isoprenaline, clearly augmented the beating rate (**Figure 3B**). In addition, rolipram (100 nM) was applied to augment any potential positive chronotropic effect of albiglutide. However, as shown in an original recording, 100 nM albiglutide, even in the presence of rolipram, did not increase the beating rate, whereas, in the same right atria, subsequently applied isoprenaline exerted a positive chronotropic effect (**Figure 3C**).

**Figure 4:**
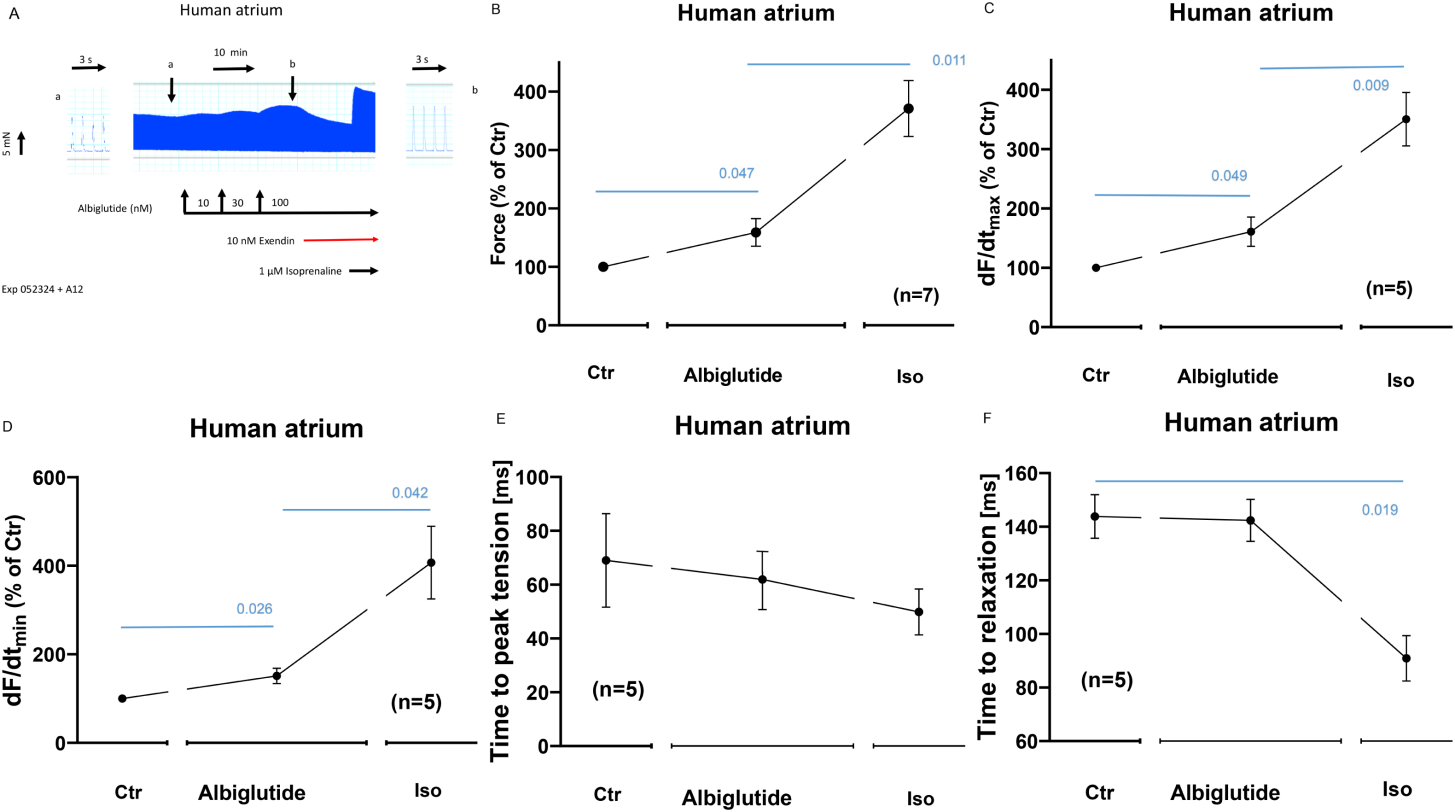
Original tracing of force of contraction over time in human atrial preparations (HAP) **(A)**. Albiglutide alone induced a time- and concentration-dependent positive inotropic effect in HAP, which was diminished by exendin(9-39) (exendin), a GLP-1R antagonist. Subsequently, a maximum effective concentration of isoprenaline (1 µM) was applied to raise the force of contraction **(A)**. At the time points labelled “a” and “b”, single contractions at higher temporal resolution were recorded, and they are depicted at the left- and right-hand sides of the main original recording. Summarised bar diagrams for the effect of 100 nM albiglutide on force of contraction **(B)**, rate of tension development [d*F*/d*t*_max_, **(C)**], rate of tension relaxation [d*F*/d*t*_min_, **(D)**], time to peak force **(E)**, and time of tension relaxation **(F)**. Ordinates are presented as mN in **(A)** and as a percentage of pre-albiglutide values in **(B–D)**. Ordinates are in milliseconds (ms) in **(E**, **F)**. The *p*-values for Albi vs. Cilo or vs. Ctr are indicated at the right-hand side of the blue line. Numbers in brackets indicate the number of experiments. Numbers (in blue) above the bars for 1 µM isoprenaline (Iso) or 100 nM albiglutide indicate significant *p*-values vs. albiglutide or vs. control values (predrug values).

Next, we studied albiglutide alone in HAP. There was a concentration- and time-dependent positive inotropic effect of albiglutide in HAP, which was reversed by 100 nM exendin(9-39), a GLP-1R antagonist. This is shown in the original recording (**Figure 4A**). We further note that 1 µM isoprenaline, when subsequently applied, had a positive inotropic effect in HAP and was more effective than 100 nM albiglutide (**Figure 4A**). To depict single contractions, we used higher temporal resolution before albiglutide (label “a” in **Figure 4A**) was applied, and after 100 nM albiglutide had reached its peak positive inotropic effect (label “b” in **Figure 4A**). Of note, albiglutide (100 nM) alone increased the force of contraction (**Figure 4B**), the rate of tension development (**Figure 4C**), and the rate of tension relaxation (**Figure 4D**). Albiglutide did not reduce the time to peak (**Figure 4E**) or the time of relaxation (**Figure 4F**). Under the same experimental conditions, 1 µM isoprenaline raised the force of contraction (**Figure 4B**), augmented the rate of tension development (**Figure 4C**), increased the rate of tension relaxation (**Figure 4D**), and shortened the time of relaxation (**Figure 4F**).

**Figure 5:**
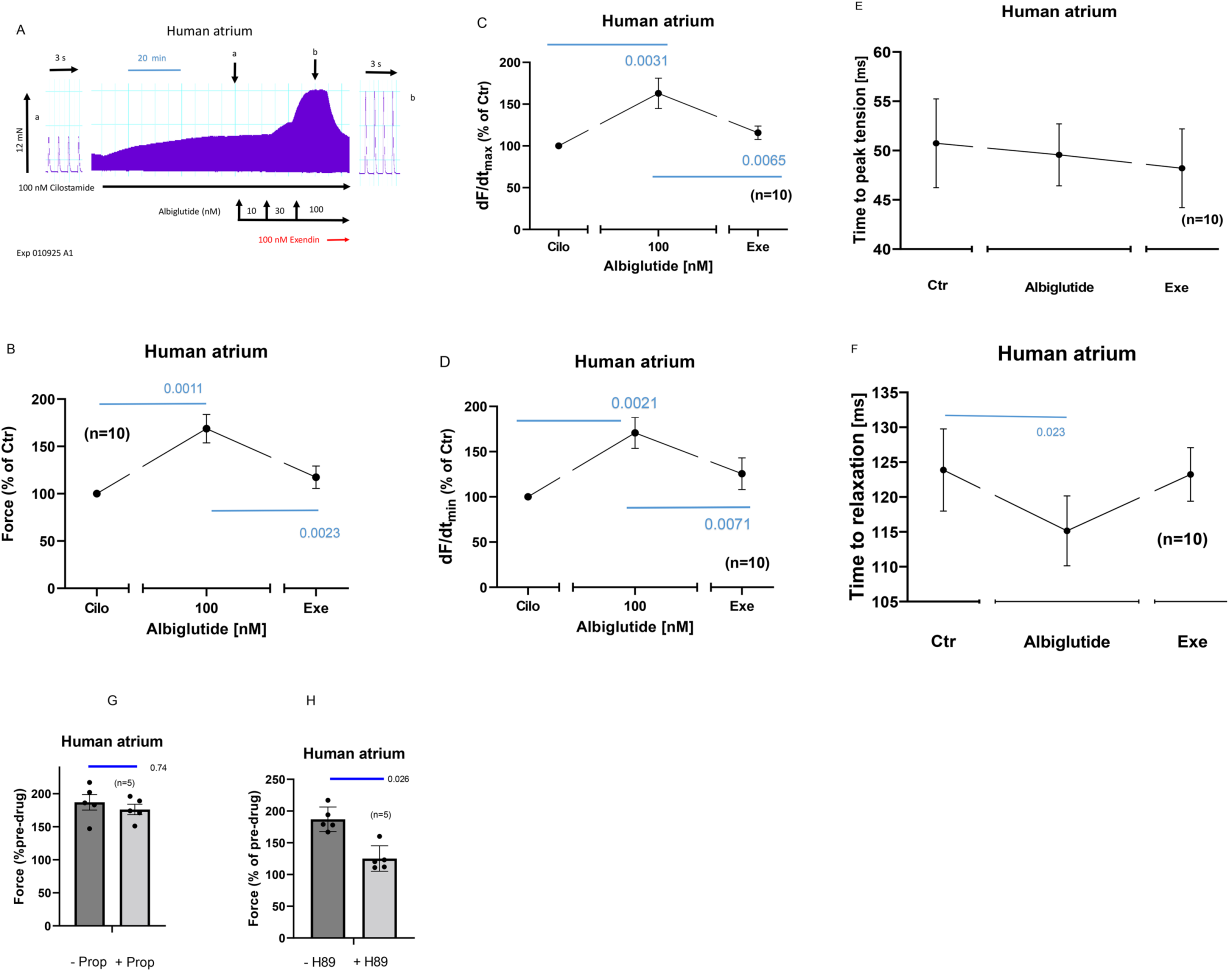
Original recordings in HAP **(A)**. In the presence of cilostamide, albiglutide induced a time- and concentration-dependent positive inotropic effect in HAP, which was diminished by exendin(9-39) (exendin), a GLP-1R antagonist **(A)**. Subsequently, a maximum effective concentration of isoprenaline (1 µM) was applied to raise the force of contraction **(A)**. At the time points labelled “a” and “b”, single contractions at higher temporal resolution were recorded, and they are depicted at the left- and right-hand sides of the main original recording. Summarised bar diagrams for the effect of 100 nM albiglutide on force of contraction **(B)**, rate of tension development [d*F*/d*t*_max_, **(C)**], rate of tension relaxation [d*F*/d*t*_min_, **(D)**], time to peak force **(E)**, and time of tension relaxation **(F)**. Ordinates are presented as a percentage of pre-albiglutide values (Ctr). Numbers (in blue) above the bars for 1 µM isoprenaline (Iso) or 100 nM albiglutide indicate significant *p*-values vs. albiglutide or vs. control values (predrug values). In **(G)**, samples were first treated with 10 µM propranolol (+Prop) or incubated alone (−Prop). Subsequently, 100 nM cilostamide and thereafter 100 µM albiglutide were added to both groups. In **(H)**, samples were first treated with 10 µM H89 (+H89) or incubated alone (−H89). Subsequently, 100 nM cilostamide and thereafter 100 µM albiglutide were added to both groups. The *p*-values for Albi vs. Cilo or vs. Ctr are indicated at the right-hand side of the blue line. Numbers in brackets indicate the number of experiments.

Next, we studied the effect of albiglutide in the presence of cilostamide. Cilostamide, a phosphodiesterase 3 inhibitor, has a well-known positive inotropic effect (**Figure 5A**). Additionally, applying albiglutide clearly raised the force of contraction. This effect increased with time after each drug addition (**Figure 5A**). The highest albiglutide concentration tested was 100 nM. When exendin(9-39), a GLP-1R antagonist, was given after 100 nM albiglutide, the force rapidly decreased, followed by a washout period (**Figure 5A**). To depict single contractions, a higher temporal resolution was used before any albiglutide (label “a” in **Figure 5A**) was given, and after 100 nM albiglutide reached its peak positive inotropic effect (label “b” in **Figure 5A**).

The data from several similar experiments on the force of contraction are summarised in **Figure 5B** and depict the positive inotropic effect of 100 nM albiglutide, which was antagonised by exendin(9-39), a GLP-1R antagonist. Furthermore, in the presence of cilostamide, 100 nM albiglutide increased the rate of tension development, and this effect was likewise antagonised by exendin(9-39) (**Figure 5C**). Moreover, in the presence of cilostamide, 100 nM albiglutide augmented the rate of tension relaxation (**Figure 5D**)—an effect antagonised by exendin (**Figure 5D**)—did not reduce muscle time to peak (**Figure 5E**), and shortened the relaxation time (**Figure 5F**). Interestingly, 10 µM propranolol did not block the positive inotropic effect of albiglutide (**Figure 5G**). We have shown that 10 µM propranolol was sufficient to block the positive inotropic effect of amphetamine, a noradrenaline-releasing agent, in HAP (Neumann et al., 2023). In contrast, H89 (an inhibitor of cAMP-dependent protein kinase) attenuated the positive inotropic effect of 100 nM albiglutide in the presence of 100 nM cilostamide in HAP (**Figure 5H**). We have previously used this concentration of H89 to attenuate GLP-1-induced positive inotropic effects in HAP (16, 31).

**Figure 6:**
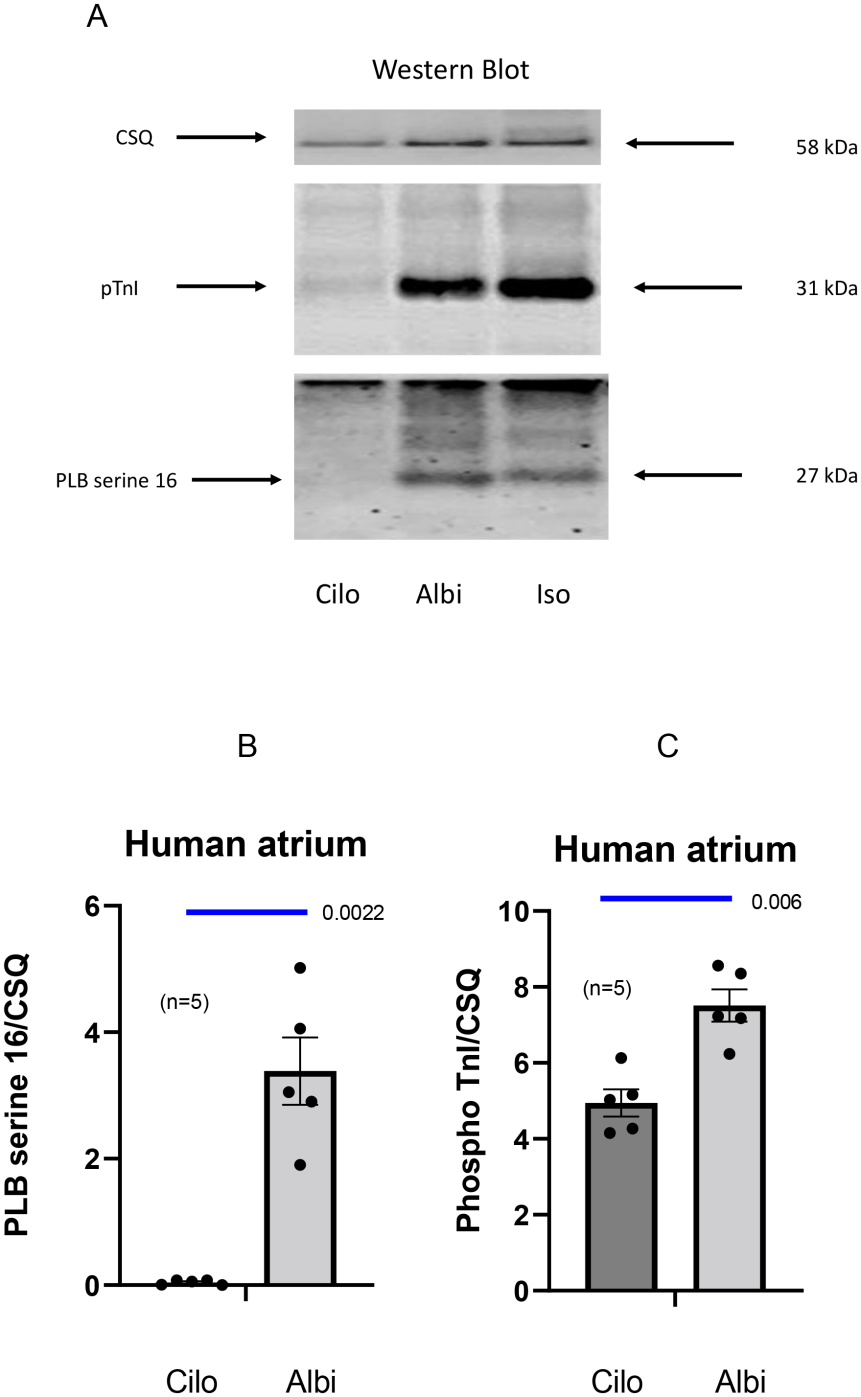
**(A)** Western blot of homogenised HAP. Samples were freeze-clamped after the addition of 100 nM cilostamide alone (Cilo), 100 nM cilostamide with 100 nM albiglutide (Albi), or cilostamide with 1 µM isoprenaline (Iso). Samples were run on a gel and transferred to nitrocellulose strips. These strips were cut horizontally and incubated first with primary antibodies and then with secondary antibodies. Depicted on the left-hand side are calsequestrin (CSQ), a cardiomyocyte-specific protein, phosphorylated troponin inhibitor (pTnI), and serine-16 phosphorylated phospholamban (PLB serine-16). On the right-hand side, molecular weights are indicated with arrows in kilodaltons (kDa). **(B)** Quantification of phosphorylated PLB. Ordinates represent the ratio of the densitometric scan of phosphorylated PLB (at amino acid sequence 16) to CSQ. An increase in phosphorylated PLB was observed when comparing samples treated with 100 nM cilostamide alone (Cilo) and 100 nM cilostamide combined with 100 nM albiglutide (Albi). The *p*-value (unpaired two-tailed *t*-test) for Albi vs. Cilo is indicated on the right-hand side of the blue line. The number in brackets indicates the number of experiments performed under each condition. **(C)** Quantification of phosphorylated TnI. Ordinates indicate the ratio of the densitometric scan of phosphorylated TnI to CSQ. An increase in phosphorylated TnI was observed when comparing samples treated with 100 nM cilostamide alone (Cilo) and those treated with 100 nM cilostamide combined with 100 nM albiglutide (Albi). The *p*-value (unpaired two-tailed *t*-test) for Albi vs. Cilo is indicated on the right-hand side of the blue line. The number in brackets indicates the number of experiments performed under each condition.

To better understand the signal transduction used by albiglutide in the human heart, we examined whether 100 nM albiglutide, in the presence of 100 nM cilostamide, would increase phosphorylation of regulatory proteins. As a negative control, we treated the HAP with only 100 nM cilostamide. Cilostamide was included in these experiments to facilitate the detection of a relevant increase in protein phosphorylation. As a positive control, the HAP was treated with 1 µM isoprenaline. Isoprenaline has been noted to increase phospholamban and TnI phosphorylation in the mammalian heart, including HAP (e.g., 32, 33). In these samples, we measured the phosphorylation state of phospholamban at serine 16 and of the inhibitory subunit of troponin (troponin I; **Figure 6**). We observed that, in this original experiment, albiglutide increased the phosphorylation state of phospholamban and the inhibitory subunit of troponin. Furthermore, we measured calsequestrin—the main Ca^2+^-binding protein of the sarcoplasmic reticulum and a marker of cardiomyocytes—to ascertain that protein content was similar after albiglutide treatment of HAP, as previously described (e.g., 33). We noted that, in the presence of 100 nM cilostamide, albiglutide (100 nM) increased the phosphorylation state of phospholamban (**Figure 6B**) and the troponin inhibitor (**Figure 6C**).

**Figure 7:**
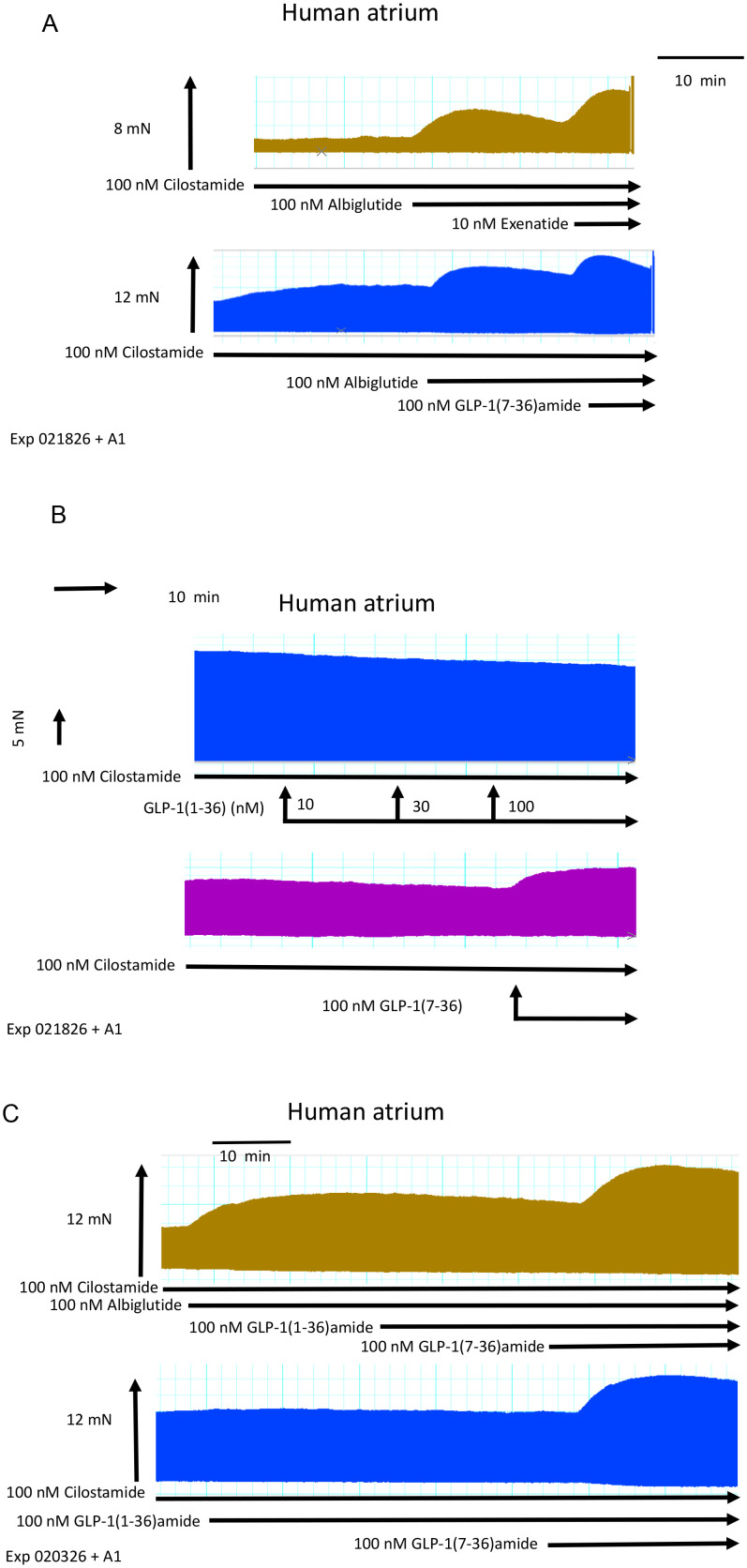
Original recordings in HAP. Albiglutide (100 nM) in the presence of 100 nM cilostamide induced a time-dependent positive inotropic effect in HAP, which was augmented by 10 nM exenatide [**(A)**, top] or 100 nM GLP-1(7-36)amide [**(A)**, bottom]. In contrast, GLP-1(1-36)amide (10, 30, or 100 nM) failed to increase force of contraction [**(B)**, top], whereas, in another muscle strip from the same patient, 100 nM GLP-1(7-36)amide raised force of contraction [**(B)**, bottom]. In a HAP from the same patient, albiglutide (100 nM) in the presence of 100 nM cilostamide induced a time-dependent positive inotropic effect. This effect was not augmented by the additional application of 100 nM GLP-1(1-36)amide [**(C)**, top]. However, when 100 nM GLP-1(7-36)amide was applied later, contractility increased [**(C)**, top]. In a different HAP from the same patient, 100 nM GLP-1(1-36)amide did not increase force of contraction in the presence of 100 nM cilostamide [**(C)**, bottom]. Nevertheless, subsequently applied 100 nM GLP-1(7-36)amide increased contractility [**(C)**, bottom]. Ordinates are presented in milliNewtons (mN). Horizontal markers indicate time in minutes (min).

Finally, we investigated whether the effect of albiglutide (100 nM) on the force of contraction can be enhanced by stimulation of GLP-1R with other endogenous or exogenous agonists. To this end, we used three GLP-1R agonists: GLP-1(7-36)amide, GLP-1(1-36)amide, and exenatide. Interestingly, in the presence of 100 nM cilostamide and 100 nM albiglutide, 10 nM exenatide increased the force of contraction (**Figures 7A**, top). This finding indicates that 100 nM albiglutide was not maximally effective in stimulating GLP-1R. Similarly, 100 nM GLP-1(7-36)amide increased the force of contraction more than 100 nM albiglutide in the presence of 100 nM cilostamide (original recording in **Figures 7A**, bottom). It is noteworthy that basal contraction values were different between preparations, conceivably due to the underlying heart diseases of the patients. One could hypothesise that full-length GLP-1(1-36)amide alone might also increase the force of contraction; however, this was not observed here (**Figure 7B**). In HAP, in the presence of 100 nM cilostamide, cumulatively applied GLP-1(1-36)amide up to 100 nM failed to increase the force of contraction (**Figures 7B**, top), whereas 100 nM GLP-1(7-36)amide did (**Figures 7B**, bottom). Next, we investigated whether GLP-1(1-36)amide would further increase the force of contraction after a prior elevation induced by 100 nM cilostamide and 100 nM albiglutide. This was not the case. Following an increase in the force of contraction induced by cilostamide and albiglutide, 100 nM GLP-1(1-36)amide did not increase the force of contraction (**Figures 7C**, top). However, the subsequent addition of 100 nM GLP-1(7-36)amide was able to further elevate the force of contraction (**Figures 7C**, top). As a control, we repeated the experiments in **Figure 7C** (top) without albiglutide. The addition of 100 nM GLP-1(1-36)amide after 100 nM cilostamide did not increase the force of contraction (**Figures 7C**, bottom). However, the addition of 100 nM GLP-1(7-36)amide increased the force of contraction (**Figures 7C**, bottom). To quantify our data, we observed that, in the presence of 100 nM cilostamide, adding 100 nM GLP-1(1-36)amide failed to raise the force of contraction (to 105% ± 7% of predrug values, *n* = 8, *p* = 0.45). In contrast, in the presence of 100 nM cilostamide, the addition of 100 nM GLP-1(7-36)amide increased the force of contraction to 145% ± 11% of predrug values (*p* = 0.029, *n* = 5). Similarly, in the presence of 100 nM cilostamide and 100 nM albiglutide, the addition of 100 nM GLP-1(7-36)amide further increased the force of contraction to 139% ± 11% of the pre-drug value (*p* = 0.044, *n* = 5). Finally, in the presence of 100 nM cilostamide and 100 nM albiglutide, the addition of 100 nM exenatide increased the force of contraction by 34% ± 9% of the predrug value (*p* = 0.043, *n* = 5).

## Discussion

The main new finding of this study is that albiglutide exerts a positive inotropic effect in HAP. These effects are mediated by GLP-1R and cAMP-dependent phosphorylation. Albiglutide acted as a partial agonist. A minor new finding was that full-length GLP-1, unlike truncated GLP-1, is unable to increase the force of contraction in isolated human atria.

The positive inotropic effects of albiglutide are probably mediated via GLP1-R, as evidenced by the reversal of its positive inotropic effect by exendin(9-39), a GLP1-R antagonist (**Figure 1**) (34). This is consistent with previous studies, including our own, on exenatide, liraglutide, and semaglutide (15, 16). GLP-1R likely increases cAMP levels in HAP. First, the positive inotropic effect of albiglutide was enhanced by cilostamide, a phosphodiesterase 3 inhibitor. Second, the putative involvement of cAMP is further supported by the finding that albiglutide increases phosphorylation at serine 16 of phospholamban. This is plausible because serine 16 is the phosphorylation site of the cAMP-dependent protein kinase in phospholamban (**Figure 1**) (19). The increased phosphorylation state of the troponin inhibitor also supports the assumption that albiglutide activates the cAMP-dependent protein kinase in HAP (**Figure 1**) (35, 36). Albiglutide can act via GLP1-R in HAP because GLP1-R is present in the human atrium (15, 16, 37, 38). It must be noted that GLP-1R is mainly—if not exclusively—located in cardiomyocytes of the human heart, placing it in the appropriate location to influence the force of contraction (39) (**Figure 1**). Atrial and ventricular positive inotropic effects of GLP-1R agonism in the human heart have previously been reported by others (15, 17, 18). In isolated, electrically driven atrial muscle preparations from 10 patients, the selective GLP-1R agonist exenatide exerted a positive inotropic effect (nonfailing atrial and ventricular preparations, 15). GLP-1(7-36)amide, the active metabolite of native GLP-1(1-36)amide, also increased the force of contraction in HAP (nonfailing atrial preparations, 15). In previous studies, we and others detected an increase in the force of contraction in HAP caused by exenatide, as well as by GLP-1 derivatives, namely liraglutide and semaglutide (16–18). In this study, we showed that albiglutide, similar to the agonist isoprenaline, increased tension rate parameters and reduced tension time parameters. This is a plausible consequence of increased phosphorylation of regulatory proteins in HAP, such as phospholamban and the troponin inhibitor.

Although 100 nM may be considered a relatively high concentration of albiglutide (40), this concentration was chosen because it convincingly increased the force of contraction in HAP. As seen in **Figure 4A**, albiglutide exhibited a positive inotropic effect at concentrations of 10 and 30 nM. It is not unusual in clinically oriented basic research to examine whether positive inotropic effects occur at supratherapeutic drug concentrations. If such effects are not observed at high concentrations, it can be reasonably concluded that a relevant positive inotropic effect is unlikely to exist. Following the basic tenets of modern pharmacology, if a positive inotropic effect is observed, it is likely to be concentration (dose)-dependent, and it can be assumed that the effect also occurs at lower concentrations, albeit with a smaller effect size.

Another justification for the use of 100 nM albiglutide is that it still allowed for further enhancement of the positive inotropic effect of albiglutide using two different GLP-1R agonists: exenatide and GLP-1(7-36)amide. Both agonists increased the force after albiglutide was applied. This indicates that GLP-1R is not maximally stimulated by 100 nM albiglutide with respect to the force of contraction. This suggests that a combination of albiglutide and another GLP-1 agonist, such as exenatide, may have additive functional effects in the heart and, conceivably, in other organs of a patient. Researchers previously reported that exenatide increases the phosphorylation state of phospholamban in HAP (15). We extend this finding to albiglutide. However, to the best of our knowledge, the present study is the first to report that any GLP-1R agonist increases the phosphorylation state of TnI. Such an increase is typical for all cAMP-increasing agents in the mammalian heart, such as isoprenaline, and is thought to contribute, at least in part, to the relaxant properties of cAMP-increasing agents in the heart (35). This report also appears to be the first to demonstrate that a GLP-1R agonist can act as a functional partial agonist in the human atrium. This may be clinically relevant for albiglutide and other GLP-1R agonists. We speculate that albiglutide, as a partial agonist, may have fewer and weaker cardiac effects. This may be either beneficial or detrimental. Albiglutide might induce fewer arrhythmias than full agonists. Others have suggested that GLP-1R stimulation might represent a novel mechanism for providing inotropic support in patients with heart failure. Such supportive effects are likely weaker for albiglutide than for other GLP-1R agonists. However, these speculations should be evaluated in future clinical trials. Interestingly, GLP-1(1-36)amide (**Figure 1**), the precursor of GLP-1(7-36)amide, failed to increase the force of contraction in HAP in the presence of cilostamide, which would be expected to amplify even small effects. One implication of this is that proteases in the human heart may degrade GLP-1(1-36)amide too slowly for any effect to be observed within the time course of our experiments. In previous studies, GLP-1(7-36)amide increased the force of contraction in HAP from nonfailing hearts obtained from cardiac transplantation programmes. However, these studies did not report any findings concerning GLP-1(1-36)amide (15). In addition, GLP-1(1-36)amide was previously shown to be virtually ineffective at stimulating cAMP formation in cells transfected with the GLP-1R receptor (41). Therefore, our data on GLP-1(1-36)amide are consistent with the literature.

### Species differences

Albiglutide did not exert a positive inotropic effect in left atrial preparations of adult mice. This finding is new but consistent with the literature. GLP-1R mRNA is present in the mouse heart. More specifically, GLP-1R mRNA is most abundant in the right atria, much lower in the left atria, and absent in both the left and right ventricles in mice (37, 42, 43). The failure of albiglutide to increase the beating rate in right atrial preparations is difficult to reconcile with the high expression of GLP1-R in the right atria of mice (37). However, a previous study found that GLP-1R is not expressed in mouse cardiomyocytes but is instead expressed in endothelial cells of the mouse endocardium (39). Hence, it is likely that albiglutide was inactive because GLP-1R is absent from the sinus node in mice.

#### Clinical relevance

Albiglutide reached a reported peak plasma concentration of 1.7 µg/mL (about 23 nM using a molecular weight of 73 kDa for albiglutide, 40). Thus, the concentrations studied in the present work can be reached in patients, highlighting the potential clinical relevance of our findings. Moreover, GLP-1R agonists (15), including albiglutide, also act on the human heart. These atrial effects might be detrimental, particularly when they induce arrhythmias; however, they may be beneficial if they lead to improved filling and, consequently, higher cardiac output (44). It must be mentioned that failure to detect a positive chronotropic effect in the mouse atrium does not imply that albiglutide exerts no positive chronotropic effect in the human atrium. In contrast, both mRNA and protein for GLP-1R have been detected in the human sinus node (45). In addition, albiglutide does not appear to have induced an indirect positive chronotropic effect, as it has been shown to increase heart rate in patients at concentrations that do not yet reduce blood pressure. A reduction in blood pressure would, via indirect extracardiac mechanisms, have triggered a compensatory increase in heart rate to maintain cardiac output constant (46).

#### Study limitations

In the patient-related part of our study, we cannot rule out the presence of atrial fibrillation or type 2 diabetes mellitus as potential confounders. This issue can be addressed in subsequent studies. Another concern is whether arrhythmias were observed in the mouse cohort. In past reports, we never detected arrhythmias in mouse left atrial preparations in our organ bath studies. In contrast, with cAMP-increasing agonists such as serotonin, isoprenaline, and dopamine in mouse right atrial preparations, we noted a higher incidence of arrhythmias compared with appropriate controls (47, 48). However, in the present study, we did not detect any arrhythmias in left or right atrial preparations of mice. One reasonable explanation is that stimulation of cAMP by albiglutide via GLP-1R does not occur in mouse cardiomyocytes, but cAMP is increased in other cells, such as fibroblasts and smooth muscle cells in the mouse atrium. Indeed, other researchers have detected GLP-1R only in noncardiomyocytes in mouse cardiac preparations (39). In contrast to mouse cardiomyocytes, the GLP-1R is present, as mRNA and protein, in human cardiomyocytes (15, 39). Whether this leads to arrhythmias should be tested in prospective clinical studies. Such studies should take into account that semaglutide has been shown to reduce the incidence of arrhythmias *in vitro* (18).

We conclude that albiglutide increases contractility in HAP via GLP-1R, activation of cAMP-dependent protein kinase, and an increased phosphorylation state of phospholamban.

## Data Availability

The original contributions presented in the study are included in the article/supplementary material. Further inquiries can be directed to the corresponding author.
